# First record of *Haematopinus tuberculatus* (Burmeister, 1839) (Psocodea: Anoplura: Haematopinidae) parasitizing buffalo (*Bubalus bubalis*) in the state of Amazonas, Brazil

**DOI:** 10.1590/S1984-29612024070

**Published:** 2024-11-22

**Authors:** José Vicente Ferreira, Marcelo Cutrim Moreira de Castro, Alexandre Levi Monteiro Santana, Gabriel Moreira Valença, André de Abreu Rangel Aguirre, Ahana Maitra, Felipe Arley Costa Pessoa

**Affiliations:** 1 Programa de Pós-graduação em Biologia da Interação Patógeno-Hospedeiro, Instituto Leônidas e Maria Deane – ILMD, Fiocruz Amazônia, Manaus, AM, Brasil; 2 Laboratório Ecologia de Doenças Transmissíveis na Amazônia, Instituto Leônidas e Maria Deane – ILMD, Fiocruz Amazônia, Manaus, AM, Brasil; 3 Laboratório de Entomologia Sistemática Urbana e Forense, Instituto Nacional de Pesquisas da Amazônia – INPA, Manaus, AM, Brasil; 4 Curso de Medicina Veterinária, Escola Superior Batista do Amazonas – ESBAM, Manaus, AM, Brasil; 5 Programa de Pós-graduação em Biologia Experimental, Fiocruz Rondônia, Porto Velho, RO, Brasil; 6 Laboratório de Entomologia, Fiocruz Rondônia, Porto Velho, RO, Brasil; 7 Department of Pharmacy-Pharmaceutical Sciences, University of Bari Aldo Moro, Bari, Apúlia, Italy

**Keywords:** Lice, Amazon, rainy season, calf, DNA amplification, Piolho, Amazônia, época chuvosa, bezerro, amplificação de DNA

## Abstract

Lice are obligate ectoparasites of birds and mammals with specialized mouthparts adapted to feed on the blood or other body tissues of their respective hosts. The registry of parasites that can cause economic and health impacts on the buffalo herd of the country is of utmost importance. In the present study, we report the first record of *Haematopinus tuberculatus* parasitizing buffalo in the municipality of Autazes, Amazonas, Brazil. The study was conducted in a rural private area located on the banks of the Paraná Madeirinha River. Twelve specimens were collected and identified as *H. tuberculatus*, comprising five females, six males, and one third instar nymph. The lice were observed on females buffalo during milking; however, the greatest abundance was found on calves, in the neck, back, and loin regions. There was no visible DNA amplification on agarose gel for the samples tested. However, the record of this louse species parasitizing on buffaloes in the region highlights the potential for epizootic outbreaks to occur in the area.

## Resumo

Piolhos são ectoparasitas obrigatórios de aves e mamíferos, possuindo peças bucais adaptadas para se alimentar do sangue ou de outros tecidos do corpo de seus respectivos hospedeiros. O registro de parasitas que possam ocasionar prejuízos econômicos e sanitários no rebanho bubalino do país é de suma importância e, por este motivo, no presente estudo foi relatado o primeiro registro de *Haematopinus tuberculatus* parasitando búfalo, no município de Autazes, Amazonas, Brasil. O estudo foi realizado em uma propriedade rural, localizada às margens do Rio Paraná Madeirinha. Foram coletados doze espécimes, identificados como *H*. *tuberculatus*, representados por cinco fêmeas, seis machos e uma ninfa de terceiro estádio. Os piolhos foram visualizados em búfalas fêmeas durante ordenha, entretanto a maior abundância foi coletada nos bezerros, na região do pescoço, dorso e lombo. Não houve amplificação de DNA visível em gel de agarose para as amostras testadas. Contudo, o registro dessa espécie de piolho parasitando búfalos no estado serve de alerta, pois surtos epizoóticos podem ocorrer na região.

Lice are insects that belong to the order Psocodea, a taxonomic group that currently encompasses the ancient orders Psocoptera and Phthiraptera (Silva-Neto et al., 2024). They are obligatory ectoparasites of birds and mammals, possessing specialized mouthparts adapted to feed on the blood or other body tissues of their respective hosts, with the majority being permanent parasites, completing their entire biological cycle on the host, unable to survive outside it (Silva-Neto et al., 2024).

Among the wide variety of parasites that can affect the health and well-being of buffaloes (*Bubalus bubalis*) in Brazil, *Haematopinus tuberculatus* (Burmeister, 1839), from the Haematopinidae family is the most commonly found louse in the country's buffalo herds (Láu, 1993). This species has been recorded in other regions, such as the states of Rio Grande do Sul (Damé, 2019), Minas Gerais (Bastianetto & Leite, 2005), São Paulo (Carvalho, 2014), Rio de Janeiro (Corrêa et al., 2012), Maranhão (Figueiredo et al., 2013), Pernambuco (Santos et al., 2010) and for the northern region (Amazon biome), in the states of Amapá (Barbosa et al., 2010), Pará (Láu, 1993; Batista et al., 2018) and Rondônia (Pinto et al., 1994).

This louse most commonly affects calves due to the abundant long hair in this age group, which facilitates the louse's movement and adherence to the host's body. Its hematophagic habit causes blood loss, and its movement over the hair leads to itching, causing ulcers, favoring secondary infections, which can even lead to the animal's death (Chaudhuri & Kumar, 1961). It is also reported as a potential vector of pathogenic agents such as *Anaplasma marginale* (Silva et al., 2013). Although its association with *Rickettsia* is unknown, this bacterium has already been detected in *Haematopinus eurysternus* parasitizing cattle in Hungary and *Haematopinus suis* parasitizing pigs in Argentina (Hornok et al., 2010; Ruiz et al., 2021).

A high infestation of *H. tuberculatus* in a buffalo herd can cause a deficit of around 30kg of live weight per animal over a period of six months. Although its impact on milk production has not yet been estimated, it is generally known that the association of these ectoparasites with their vertebrate hosts directly affects the overall productivity of the herd (Láu, 1993).

Buffalo farming in Brazil holds a prominent position on the world stage, with 1,598,268 registered animals (IBGE, 2022). Pará is the largest producer in the country, with 644,672 animals. The Marajó Archipelago is the main center of buffalo production in the region. Amapá has 312,355 buffaloes, and the state of São Paulo has the third-largest herd, with 122,766 heads (IBGE, 2022).

Amazonas holds fourth place in the national ranking with 113,557 buffaloes, with the municipality of Autazes being the largest breeder in the state, with a herd of 45,572 animals (IBGE, 2022). Popularly known as the land of milk and a pioneer in the country in the form of “floating cheese factories” with State Inspection Service, it is responsible for supplying the capital of Amazonas with the production of coalho-type buffalo cheese, fresh minas, and mozzarella (IDAM, 2015).

The municipalities of Itacoatiara, Nova Olinda do Norte, and Careiro da Várzea make up the largest dairy basin in the state of Amazonas, together with the municipality of Autazes, which is currently the largest producer of bovine and buffalo milk (IBGE, 2022). In 2017, it was considered the municipality with the largest buffalo milk production in Brazil, with 9.3 million liters (IBGE, 2017).

Given this scenario, it is extremely important to record the occurrence of parasites that can cause economic and health losses in the country’s buffalo herd. Such records will help to understand their prevalence and geographic dispersion patterns. Therefore, in this study, we report the first record of *H. tuberculatus* parasitizing buffalo in the municipality of Autazes, state of Amazonas, Brazil.

The municipality of Autazes is located in the metropolitan region of Manaus, in the state of Amazonas. It is characterized by several rivers and lakes, and features a humid tropical climate (Type Af), according to the Köppen classification. It is located at an altitude of approximately 23m, with temperatures ranging from 24 °C to 34 °C, and experiences precipitation throughout the year (Weatherspark, 2024).

**Figure 1:**
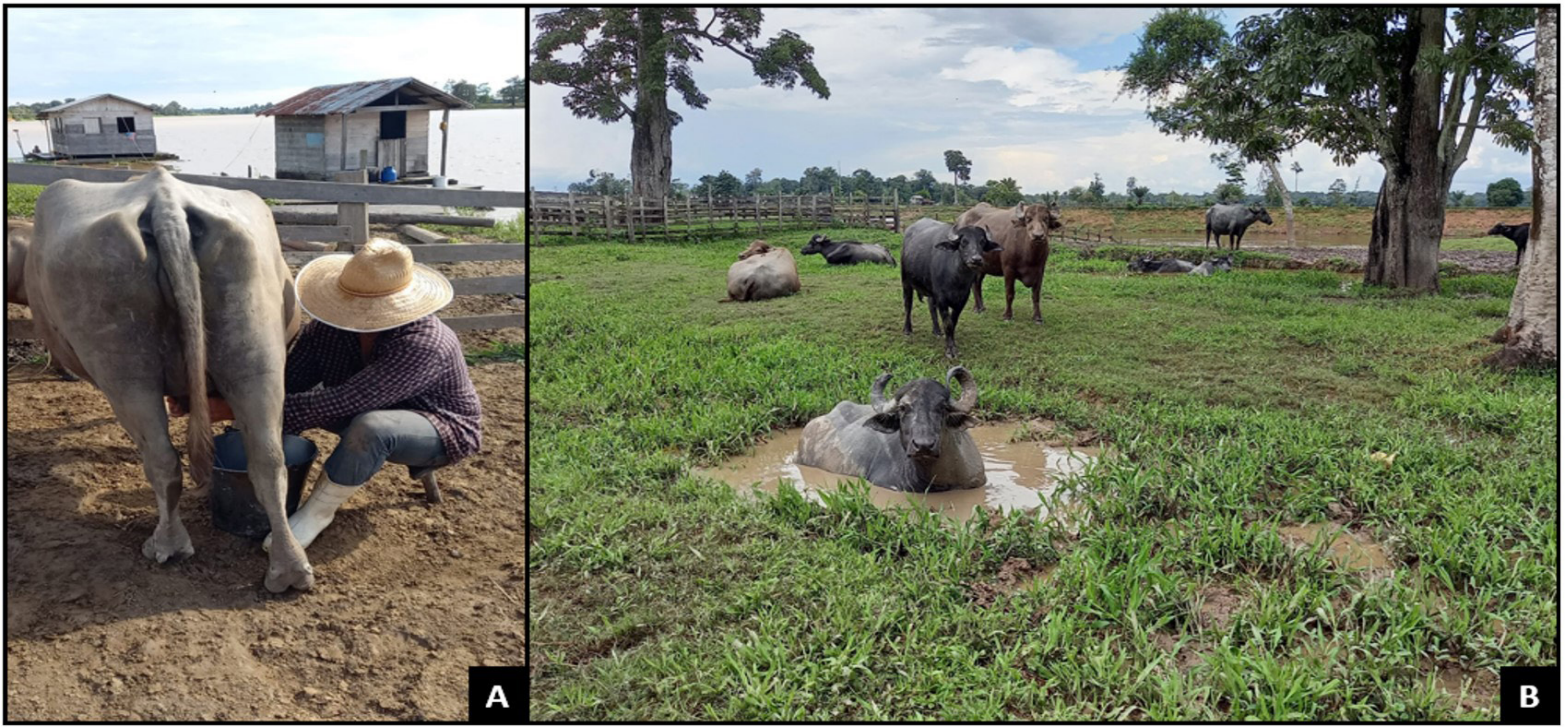
Cowboy milking buffalo in the corral (A); Buffalo herd on the farm (Amazon biome) in a floodplain area (B).

The study was conducted on a rural property (03°50’59” S 59°39’28” W) located on the banks of the Paraná Madeirinha River. The property encompasses floodplain ecosystems, igapó forest, and dense *terra firma* rainforest, with pastures mainly composed of grasses like *Brachiaria humidicula* and *Brachiaria mutica*. The herd comprises 48 buffaloes, specifically of the Mediterranean and Jafarabadi breeds (Figure 1).

A preliminary inspection was carried out on the lactating buffalo herd at the property, and subsequently, a systematic manual collection was conducted randomly with the aid of entomological forceps. The lice specimens were carefully placed in 1.5 mL microtubes containing absolute ethanol and duly labeled. After the collection, the samples were transported to the Urban Systematic and Forensic Entomology Laboratory of the National Institute for Amazonian Research.

For species-level identification, the insect specimens were mounted on a slide with Canada balsam following identification keys (Werneck, 1936). Images of specimens were obtained with a Leica DFC500 digital camera coupled to a Leica M205c stereoscopic microscope, connected to a computer with the Leica Application Suite LAS V3.6 software, which includes a self-assembly module. Following identification, the lice specimens were stored in a microtube containing absolute ethanol for subsequent molecular biology analyses.

DNA samples from lice were individually obtained from each specimen using an extraction protocol with guanidine isothiocyanate and phenol, as described by Sangioni et al. (2005) and modified by Matrone et al. (2009). The samples were quantified and diluted to a final concentration between 20 and 30 ng/µL. Two polymerase chain reactions (PCRs) were performed for each specimen: one aiming to amplify fragments of the *gltA* genes, of 401 base pairs (bp), specific for the *Rickettsia* genus, and another targeting a 360 bp fragment of *16S* rRNA with primers specific for the Anaplasmataceae family. The primers to amplify *gltA* were CS78 5'- GCAAGTATCGGTGAGGATGTAAT-3' and CS322 5'- GCTTCCTTAAAATTCAATAAATCAGGAT-3' (Labruna et al., 2004). For *16S* rRNA amplification, the primers used were GE2 5'- GTTAGTGGCAGACGGGTGAGT-3' and GE3 5'- TATAGGTACCGTCATTATCTTCCCTAT-3' (Breitschwerdt et al., 1998). PCR products were visualized on a 1.5% agarose gel.

**Figure 2:**
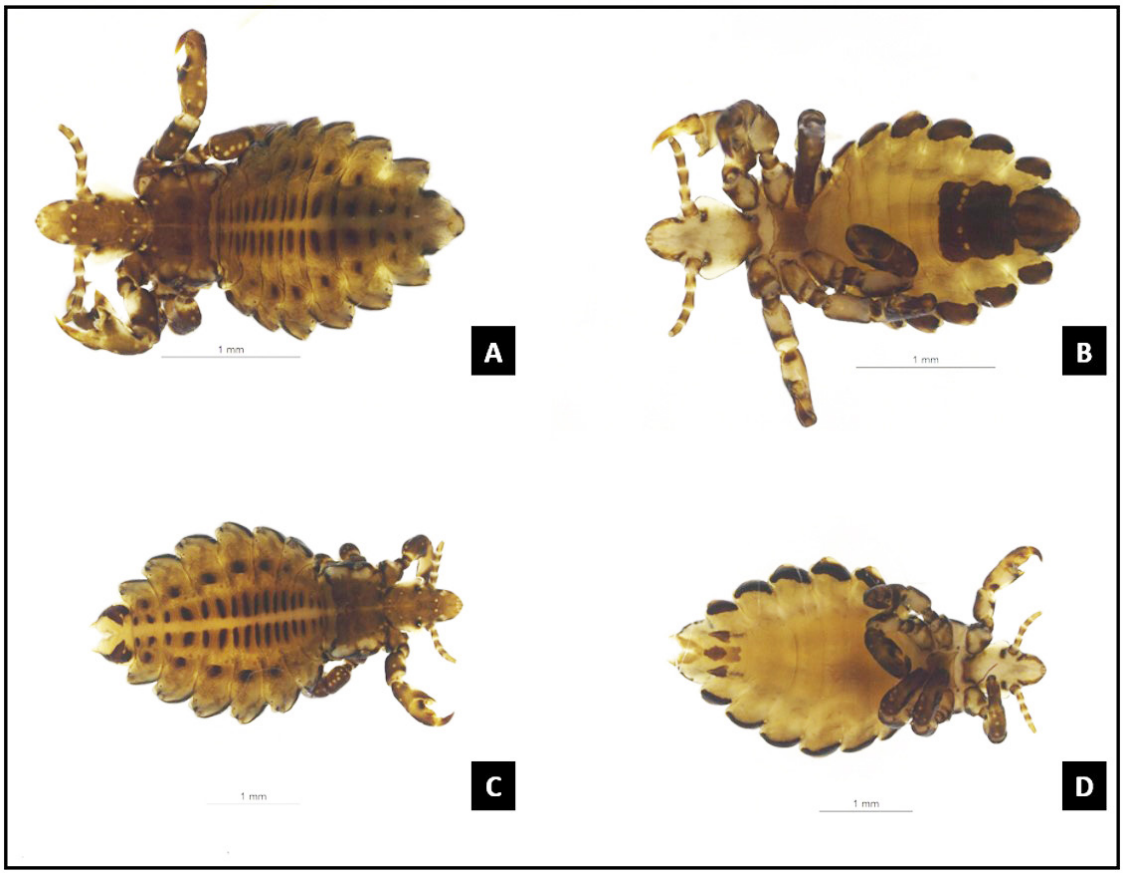
Microphotograph of *Haematopinus tuberculatus*. Male dorsal view (A); Male ventral view (B); Female dorsal view (C); Female ventral view (D).

Twelve lice specimens were collected for the first time in the state of Amazonas during the region's rainy season (January 2022). The specimens, comprising five females, six males, and a third nymph stage, were collected from the corral of a rural property and were identified as *Haematopinus tuberculatus* (Figure 2).

*Haematopinus tuberculatus* is a common species in buffalo farms, having already been recorded in other regions of the country; however, most studies are restricted to the north and southeast regions, as these regions have the concentration of the largest number of buffaloes.

In the present study, lice were observed on females buffalo during milking; however, the greatest abundance was collected from calves. This finding corroborates the results of Chaudhuri & Kumar (1961), who also demonstrated that calves are more susceptible to higher infestations of these parasites. Their coats are more evident than in adult animals, facilitating the parasite's movement and adherence to the host.

The lice were predominantly observed on the neck, back, and loins, corroborating the findings from previous studies of Figueiredo et al. (2013) in Maranhão and Batista et al. (2018) in Pará, where preferences for similar anatomical regions had been observed. This behavior in the present study may be attributed to the fact that these areas are more exposed to the water's surface during the thermoregulation of buffaloes, which may be associated with greater hair accumulation in these areas.

There was no visible DNA amplification in agarose gel for the samples tested for the *gltA* and *16S* rRNA gene fragments of Anaplasmataceae, indicating that no lice were associated with *Rickettsia*, *Anaplasma*, and *Ehrlichia*.

The lack of studies on the prevalence of parasites of veterinary importance in the buffalo herd and the record of a new species of louse parasitizing buffaloes in the state serve as a caution to breeders and veterinarians. The potential probability of epizootic outbreaks influenced by this ectoparasite in an economically crucial region for dairy farming emphasizes the utmost need and importance of efficient monitoring and surveillance entomological practices.
